# Altered leptin signaling and attenuated cardiac vagal activity in rats with type 2 diabetes

**DOI:** 10.3389/fphys.2025.1547901

**Published:** 2025-02-26

**Authors:** Anthony J. Evans, Huiyin Tu, Yu Li, Boris Shabaltiy, Lauren Whitney, Kassidy Carpenter, Yu-long Li

**Affiliations:** ^1^ Department of Emergency Medicine, University of Nebraska Medical Center, Omaha, NE, United States; ^2^ Department of Cellular & Integrative Physiology, University of Nebraska Medical Center, Omaha, NE, United States

**Keywords:** cardiac parasympathetic activity, intracardiac ganglia, leptin resistance, type 2 diabetes, uncoupling protein 2

## Abstract

**Introduction:**

The leading cause of death in type 2 diabetes mellitus (T2DM) patients is cardiovascular-related events, including myocardial infraction-induced ventricular arrhythmia. Previous studies have shown that T2DM-induced functional remodeling of cardiac vagal postganglionic (CVP) neurons contributes to ventricular arrhythmogenesis. As leptin resistance is common in T2DM patients, and CVP neurons are located in epicardial adipose pads, a tissue that secretes leptin, in this study we aimed to elucidate a correlation between leptin resistance and CVP neuronal dysfunction in T2DM.

**Methods:**

A high fat-diet/low dose streptozotocin-induced T2DM rat model was used in this study to characterize T2DM-induced alterations in cardiac parasympathetic tone, determined by changes in baroreflex sensitivity and CVP neuronal excitability. The impact of leptin resistance on CVP neurons was also studied by examining the expression of leptin in epicardial adipose pads, and leptin receptors and uncoupling protein 2 (UCP2) in CVP neurons.

**Results:**

T2DM rats exhibited diminished baroreflex sensitivity, and decreased CVP neuronal excitability, demonstrated by a reduced frequency of action potentials, diminished nAChR currents, and an attenuated response to nicotine stimulation. Additionally, compared to sham animals, the expression of leptin receptors and UCP2 in CVP neurons was reduced as early as 4 weeks post-T2DM although the leptin levels in epicardial adipose pads was increased during the progression of T2DM, which demonstrated the occurrence of leptin resistance in T2DM CVP neurons.

**Conclusion:**

Cardiac parasympathetic dysfunction in T2DM rats is due, in part, to functional remodeling of CVP neurons. As leptin resistance develops as early as 4 weeks post-T2DM induction, diminished leptin receptors-UCP2 signaling may contribute to CVP neuronal dysregulation.

## Introduction

Type 2 diabetes mellitus (T2DM) is a common metabolic disease that impacts more than 500 million individuals worldwide, with an estimated healthcare cost anticipated to exceed $1054 billion by the year 2045 (International Diabetes Federation, 2021; Hossain et al., 2024). The leading cause of mortality in the T2DM population is cardiovascular-related events including stroke, myocardial infarction (MI), and sudden cardiac death (SCD) (Walker and Cubbon, 2015; Rawshani et al., 2017; Gyldenkerne et al., 2023; Wong and Sattar, 2023). The development of ventricular tachycardia/ventricular fibrillation (VT/VF) post-MI has been linked with sudden cardiac death in T2DM patients, with ventricular arrhythmias accounting for one-third of abnormal heart rhythms observed at the time of cardiac arrest (Agarwal and Singh, 2017; Mhaimeed et al., 2023; Chakraborty et al., 2024). While tight glycemic control is the typical treatment for many diabetes-related pathologies, multiple studies have reported that the risk of cardiovascular event-related deaths in T2DM patients is not reduced by glycemic control alone (Reaven et al., 2019; Scheen, 2022).

A significant contributor to ventricular arrhythmogenesis in T2DM is the remodeling of the parasympathetic division of the intrinsic cardiac nervous system which exerts autonomic control of the heart (Zhang et al., 2018; Hu et al., 2021). The efferent component of the cardiac parasympathetic nervous system begins with preganglionic parasympathetic neurons that extend from the dorsal vagal motor nucleus (DVMN) and nucleus ambiguus through the left and right cardiac trunks of the vagus nerve (Armour, 2007; Durães Campos et al., 2018). The latter ultimately innervates cardiac vagal postganglionic (CVP) neurons located in the intracardiac ganglia (ICG) embedded in the epicardial adipose pad on the heart (Armour, 2007; Durães Campos et al., 2018). Our previous work has shown that CVP neurons in T2DM exhibit reduced nicotinic acetylcholine receptor (nAChR) currents and N-type voltage-gated calcium currents, which result in compromised neuronal function and increased susceptibility to arrhythmogenesis (Liu et al., 2012; Liu et al., 2015; Zhang et al., 2022). The molecular mechanisms driving changes in CVP neuronal function as T2DM develops have not yet been elucidated.

Leptin resistance, as defined by reduced sensitivity to the hormone leptin, is a common complication of T2DM (Schmidt et al., 2006; Gruzdeva et al., 2019). Leptin, predominantly secreted by adipose tissue, binds leptin receptors on the cell membrane, which canonically activates the Janus kinase/signal transducer and activator of transcription (JAK-STAT) pathway (Procaccini et al., 2009). Previous studies have reported on leptin signaling’s role in modulating neuronal excitability in Pro-opiomelanocortin (POMC) neurons (Perissinotti et al., 2021), striatal cholinergic interneurons (Mancini et al., 2022), and neuropeptide Y (NPY)/Agouti-related protein (AgRP) neurons of the hypothalamic arcuate nucleus (Takahashi and Cone, 2005). Currently, the impact of leptin resistance on CVP neuronal function has not yet been explored. As the ICG are located in epicardial adipose, the proximity of CVP neurons to epicardial adipocytes speaks to a potential role for leptin signaling in CVP neuronal function. We hypothesized that CVP neurons would develop leptin resistance as T2DM progresses, which further contributes to T2DM-induced CVP neuronal dysregulation. Therefore, this study aimed to further characterize the impact of T2DM on cardiac parasympathetic function and explore the potential role of leptin resistance in CVP functional remodeling.

## Materials and methods

### Animal studies

This study conforms to guidelines for the Care and Use of Laboratory Animals and was approved by the Institutional Animal Care and Use Committee (IACUC) at the University of Nebraska Medical Center (IACUC number: 23-043-09-FC and 18-023-04-FC). After *in vivo* experiments were completed, rats were euthanized via i.p. injection of 0.39 mL/kg of Fatal-Plus euthanasia (about 150 mg/kg pentobarbital, DBA Med-Vet International, Mettawa, IL, United States).

### T2DM rat model

Male and female Sprague-Dawley rats (200–220 g) were housed in a controlled temperature and humidity environment and were exposed to a 12-h light to 12-h dark cycle. Water and chow were provided *ad libitum.* Sham rats were administered a standard chow diet (34% protein, 53% carbohydrate, 13% fat). T2DM was induced via introduction of a high-fat diet (HFD) (15.2% protein, 42.7% carbohydrate, 42% fat; Inotiv Pharmaceutical Company, Indianapolis, IN, United States). After 4 weeks of HFD introduction, rats were injected with a low dose of streptozotocin (STZ) (30 mg/kg, i.p.) and continued HFD feeding until sacrifice at different time-points. Fasting blood glucose was measured every 4 weeks. All experiments were performed after 4, 8, or 12 weeks on standard chow or HFD. Metabolic characteristics of rats used in this study are summarized in Supplementary Table 1. Sham rats are defined in this study as rats that did not receive HFD or STZ treatment. Sham rats used were a mix of rats age-matched to the 4, 8, and 12-week experimental groups. We chose a mixed control group as we have not observed any difference in the parameters measured in this study among sham rats of different ages.

### Measurement of baroreflex sensitivity

A combination of chloralose (40 mg/kg, i.p.) and urethane (800 mg/kg, i.p.) was used to anesthetize rats, with an additional supplementation of chloralose (10 mg/kg, i.v.) every 2 h. A ventral midline neck incision was made, the trachea was cannulated, and artificial ventilation was performed via mechanical respirator (Harvard Apparatus, Holliston, MA, United States; 60 breaths/min; 2.5 mL tidal volume). A Millar pressure transducer (SPR 524, ADInstruments, Colorado Springs, CO, United States) was inserted into the right carotid artery for testing arterial blood pressure (ABP) and heart rate (HR). A polyethylene catheter was implanted into the right femoral vein for drug administration.

Baroreflex sensitivity was determined by measuring reflex changes in HR responding to changes in ABP. Sodium nitroprusside (30 μg, i.v.) was administered to decrease ABP to approximately 50 mmHg. ABP was subsequently raised through administration of phenylephrine (10 μg, i.v.). The logistic regression curve was then fit using the equation: $HR=A/1+e^{B\left(blood\;pressure-C\right)}+D$ (A = HR range, B = Slope coefficient, C = ABP at midpoint of range, D = minimum HR) (Zhang et al., 2014). The maximum gain, an index of baroreflex sensitivity, was determined by calculating A*B/4 of the logistic regression curve.

### Isolation of CVP neurons and whole cell patch-clamp recording for nAChR currents and action potentials

CVP neurons were isolated by a two-step enzymatic digestion protocol (Liu et al., 2012). Briefly, collected ICG was minced into small pieces and incubated successively with digestion solution one containing 0.1% collagenase and trypsin, and digestion solution two containing 0.2% collagenase and 0.5% bovine serum albumin for 30 min at 37°C. The isolated neurons were cultured in the incubator for patch clamp recording or immunofluorescence staining.

Action potentials and nAChR currents were recorded by the whole cell patch-clamp technique using an Axopatch 200B patch-clamp amplifier (Axon Instruments, Foster City, CA, United States). The P-clamp 10.2 program (Axon Instruments) was used for data acquisition and analysis. Recorded traces were sampled at 10 kHz and filtered at 5 kHz. All experiments were done at room temperature (22°C–24°C).

The nAChR currents were recorded by a gap-free voltage-clamp technique. The resistance of the patch pipette was 3–5 MΩ when pipette was filled with the following solution (in mM): 140 CsCl, 2 MgATP, 10 HEPES, and 1 EGTA (pH 7.2, 300 mOsm/L). The extracellular solution consisted of (in mM): 140 NaCl, 3 KCl, 2.5 CaCl_2_, 1.2 MgCl_2_, 10 HEPES, and 7.7 glucose (pH 7.2; 310 mOsm/L). Series resistance of 6–13 MΩ was electronically compensated to 80%–90%. Junction potential was calculated to be +5.1 mV. CVP neurons were clamped at −90 mV and continuously perfused with extracellular solution (2 mL/min). Nicotine, a nAChR specific agonist dissolved in the extracellular solution, was applied to ICG neurons by bolus ejection (6 psi.; 100 m) through a micropipette connected to a Picopump (PV 820 Pneumatic PicoPump, World Precision Instruments, Sarasota, FL). The micropipette (5–7 µm diameter) for drug application was positioned 20–30 µm from the cell soma. After nicotine concentration (3 mM) was applied (Liu et al., 2015), peak currents were measured, and current density was calculated by dividing peak current by cell membrane capacitance (C_m_).

In the current-clamp model, frequency of action potentials was measured in a 1-s current clamp (100 pA). The patch pipette solution consisted of (in mM): 105 K-Aspartate, 20 KCl, 1 CaCl_2_, 5 MgATP, 10 HEPES, 10 EGTA, and 25 glucose (pH 7.2; 320 mOsm/L). The bath solution was composed of (in mM): 140 NaCl, 5.4 KCl, 0.5 MgCl_2_, 2.5 CaCl_2_, 5.5 HEPES, 11 glucose, and 10 sucrose (pH 7.4; 330 mOsm/L). Junction potential was calculated to be +12.3 mV and membrane potential was corrected using this value. Based on our preliminary data and one previous study (Alkondon et al., 2000), 30 µM nicotine was perfused to measure resting membrane potential and frequency of action potentials.

### Reverse-transcriptase quantitative polymerase chain reaction (RT-qPCR)

RNA was isolated from rat ICG using Trizol reagent (Thermofisher, Walham, MA, United States) following the manufacturers protocol. Five-hundred ng of total RNA was subsequently reverse-transcribed (RT) using the iScript Reverse Transcription Supermix (Bio-rad laboratories, Hercules, CA, United States). RT was performed at 42°C for 30 min. Quantitative PCR was performed using iQ Sybr-green supermix (Bio-rad laboratories, Hercules, CA, United States) using 1 µL of cDNA as template. PCR reactions were cycled in the Step-one plus real time PCR system (Applied Biosystems, Foster City, CA, United States). PCR was performed using the following cycling parameters: 1 cycle of 95°C for 10 min, 40 cycles of 95°C for 15 s, followed by 60° for 1 min. Following the end of cycling, a melting curve analysis was performed. For quantification, each gene of interest was normalized to the housekeeping gene specified in the corresponding figure legend. The data were analyzed using the 2^−ΔΔCt^ method. All primers used for amplification are listed in Supplementary Table 3.

### Reverse Phase protein Microarray (RPPA)

Due to the limitation of small ICG samples (1–2 mg wet weight), we could not detect the protein expression using regular Western blot analysis and instead employed a modified RPPA as previously described (Zhang et al., 2018). In short, ICG were rapidly removed, flash frozen, and stored at −80°C. ICG proteins were extracted in protein lysis buffer (10 mM Tris, 1 mM EDTA, 1% SDS; pH 7.4) plus protease inhibitor (Sigma, St. Louis, MO, United States) and incubated on ice for 1 h. Homogenates were centrifuged at 12,000×g for 30 min at 4°C and the supernatants were harvested. Protein concentrations were determined using bicinchoninic acid (BCA) assay (Thermofisher, Waltham, MA, United States) and proteins were standardized to equal concentrations. 50 nL of each protein homogenate was loaded onto nitrocellulose-coated glass slides using an 8-pin arrayer. Nitrocellulose slides were subsequently blocked using Intercept blocking buffer (LI-COR, Lincoln, NE, United States) for 1 h at room temperature while shaking. Slides were then incubated in primary antibody (Supplementary Table 2) overnight shaking at 4°C. Slides were washed 3X in TBS-T for 10 min at room temperature and incubated with appropriate fluorescence-conjugated secondary antibodies for 1 h at room temperature. Slides were subsequently washed 3X in TBS-T for 10 min and visualized using the LI-COR Odyssey DLX imaging system (Li-COR, Lincoln, NE, United States). Protein expression was quantified using ImageJ analysis software (NIH, Bethesda, MD, United States).

### Immunofluorescence

Isolated CVP neurons were plated onto slides and were subsequently fixed with 4% paraformaldehyde in 0.1 M PBS for 10 min at 4°C, washed with PBS-T, and blocked with 10% normal goat serum for 1 h at room temperature. CVP neurons were incubated with primary antibodies listed in Supplementary Table 2 overnight at 4°C. Slides were washed and subsequently incubated with appropriate secondary antibodies for 1 h at room temperature. CVP neurons were observed under a Leica fluorescent microscope with appropriate excitation/emission filters (Leica Microsystems, Buffalo Grove, IL, United States). Pictures were captured by a digital camera system. No staining was observed when PBS was used in place of primary antibody. Protein expression was quantified using Adobe Photoshop (Adobe Systems).

### Western blot analysis

Rat epicardial adipose pads were rapidly removed and flash frozen until used. Rat epicardial adipose pads were homogenized in RIPA buffer (50 mM Tris-HCl, 150 mM NaCl, 50 mM NaF, 2 mM EDTA, 1 mM Na_3_VO_4_, 1%NP-40, 1%SDS, 1 mM PMSF) supplemented with a protease inhibitor cocktail (Sigma, St. Louis, MO, United States) using the bead blaster homogenization system (Benchmark Scientific, Sayreville, NJ, United States). Homogenates were incubated on ice for 1 h and then centrifuged (12,000×g at 4°C for 30 min) to remove excess tissue debris. Protein concentrations were determined using a BCA assay, and concentrations were standardized across all samples. 50 μg of total protein was loaded onto 13% SDS-PAGE gels and run at 100 V for 1 h at room temperature. Proteins were subsequently transferred to PVDF membranes via wet transfer method for 1.5 h at 100 V. PVDF membranes were incubated in ponceau-s solution (Thermofisher, Waltham, MA, United States) to visualize total protein content of each sample. Membranes were washed 3X in TBS-T for 10 min at room temperature and subsequently blocked in Intercept blocking buffer (LI-COR, Lincoln, NE, United States) for 1 h at room temperature. Blots were incubated in primary antibody (Supplementary Table 2) overnight at 4°C while rotating. Blots were subsequently washed 3X in TBS-T for 10 min at room temperature and probed with appropriate fluorescence-conjugated secondary antibodies for 1 h at room temperature. Blots were washed in TBS-T 3X for 10 min and visualized using the LI-COR Odyssey DLX imaging system (LI-COR, Lincoln, NE, United States). Protein expression was quantified using ImageJ analysis software (NIH, Bethesda, MA, United States).

### Statistics

It has been previously reported that the pathogenesis of T2DM in patients exhibits sexual dimorphism (Kautzky-Willer et al., 2023). To determine if there was sex-based variation in CVP neuronal function, we examined nAChR currents and action potential frequency in CVP neurons of 12-week T2DM male and female rats. No significant difference in nAChR currents or action potential frequency in CVP neurons was observed between male and female rats in either the sham or T2DM condition (Supplementary Figures 1A, B). As we did not find sex differences in any parameters in the present study, we mixed sexes for statistical analyses.

Statistical analysis was performed using GraphPad Prism software (GraphPad software inc., La Jolla, CA, United States). All data are presented as mean ± SEM. An unpaired t-test or a one-way ANOVA with *post hoc* Dunnetts test was used to determine significance. Statistical significance was accepted when P < 0.05.

## Results

### Induction of T2DM

A previously described (Liu et al., 2012; Hu et al., 2021) HFD and a low-dose STZ rat model was used in this study because rats treated for 12 weeks with HFD and one-time low-dose STZ injection (deemed T2DM rats in this study) closely mimic the clinical characteristics, such as reduced insulin sensitivity, dyslipidemia, and hyperglycemia, seen in T2DM patients (Zhang et al., 2008; Liu et al., 2012; Skovsø, 2014; Cui et al., 2018). At 8 and 12 weeks post-T2DM induction, elevated levels of fasting blood glucose were observed in T2DM rats as compared to sham, indicating that HFD-STZ treatment induced a hyperglycemic state as early as 8 weeks post-T2DM induction (Supplementary Table 1). However, no significant difference was found in the body weight for T2DM and sham rats at any timepoint studied (Supplementary Table 1).

### T2DM reduced baroreflex sensitivity

**FIGURE 1:**
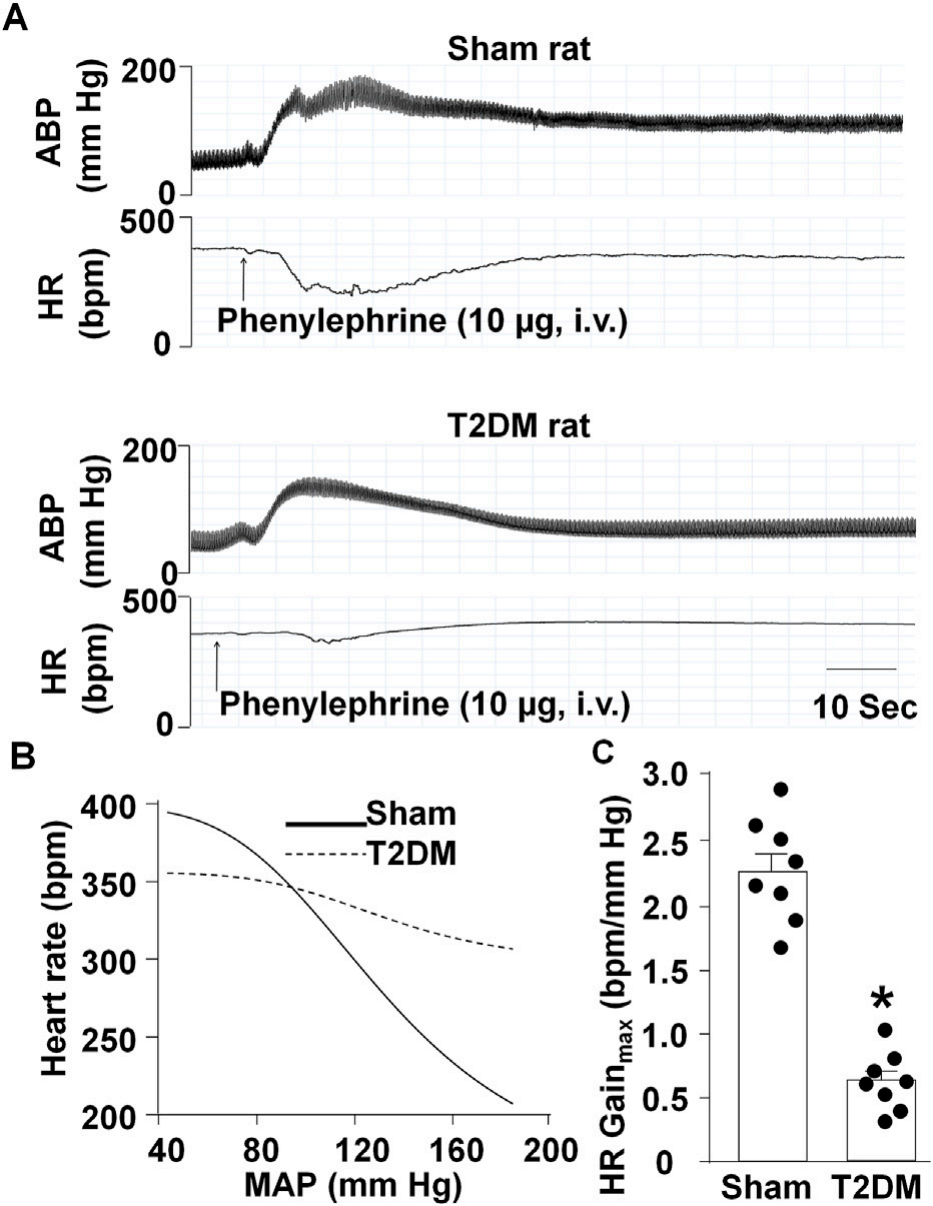
Arterial baroreflex sensitivity in sham and T2DM rats. **(A, B)** Representative recordings **(A)** and logistic regression curves **(B)** for reflex changes in heart rate (HR) in response to changes in arterial blood pressure (ABP) in anesthetized sham and T2DM rats. **(C)** Quantitative data for maximum gains of HR calculated from logistic regression curves. Arterial blood pressure was decreased to about 50 mmHg by sodium nitroprusside (30 μg, i.v.) and then increased by phenylephrine (10 μg, i.v.). Data are mean ± SEM, n = 8 rats in each group. *P < 0.05 vs. sham.

To explore the impact of T2DM development on cardiac autonomic function, we examined baroreflex sensitivity. Baroreflex sensitivity was determined by measuring changes in HR in response to pharmacologically induced changes in ABP in sham and 12-week T2DM rats (Figures 1A,B). 12-week T2DM rats exhibited a significantly lower HR Gain_max_ (an index of baroreflex sensitivity) as compared to sham rats (Figure 1C), indicating that T2DM development results in diminished baroreflex sensitivity.

### T2DM diminished CVP neuronal excitability

**FIGURE 2:**
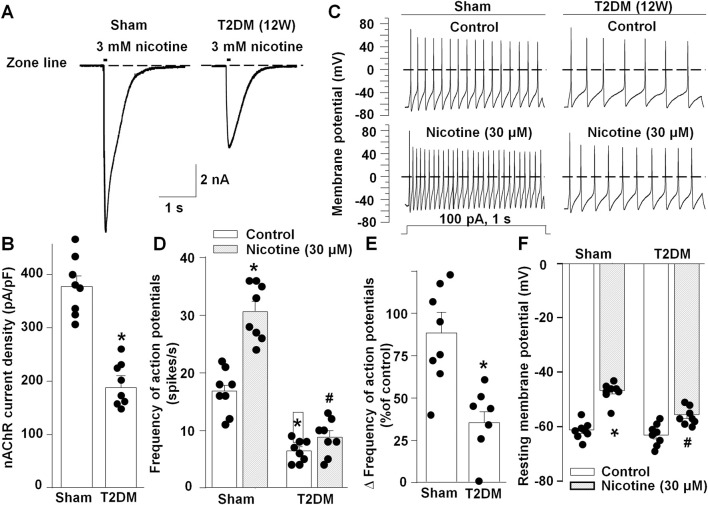
nAChR currents and cell excitability in CVP neurons from sham and T2DM rats. **(A, B)** Original recordings **(A)** and mean data **(B)** of nicotine (3 mM, 100 m)-evoked nAChR currents. **(C, D)** Original recordings of the action potential **(C)** and frequency of action potentials **(D)** before and after treatment with nicotine (30 µM) in CVP neurons. **(E)** Percent change of action potential frequency in response to nicotine in CVP neurons. **(F)** Resting membrane potentials before and after treatment with nicotine (30 µM) in CVP neurons. Data are mean ± SEM, n = 8 neurons in each group. *P < 0.05 vs. sham; ^#^P < 0.05 vs. T2DM control.

Baroreflex sensitivity is regulated at multiple centers, including the efferent limb that is, in part, controlled by parasympathetic activity of the vagus nerve (Kuo et al., 2005; Dall’ago et al., 2007; Soltani et al., 2023). Furthermore, it has been reported that the vagus nerve becomes structurally and functionally compromised during T2DM progression (Guo et al., 1987; Lee et al., 2002; Dall’ago et al., 2007; Ziegler et al., 2018; Evans and Li, 2024). To explore the effect of T2DM on cardiac vagal activity, we assessed the impact of T2DM development on CVP neuronal function. In CVP neurons from sham and 12-week T2DM rats, 3 mM nicotine-induced nAChR currents were measured. A significantly lower nAChR current density was observed in CVP neurons from 12-week T2DM rats compared to sham rats, consistent with previously reported results (Liu et al., 2015) (Figures 2A,B).

To assess if reduced nAChR current densities impact CVP neuronal excitability, we recorded action potentials in isolated CVP neurons from sham and 12-week T2DM rats and measured the frequency of action potentials with or without nicotine treatment (30 µM). CVP neurons from T2DM rats exhibited fewer action potentials compared to that from sham rats (Figures 2C,D). Additionally, nicotine-increased action potential frequency was severely blunted in T2DM CVP neurons as compared to sham CVP neurons (Figures 2D,E). Furthermore, there is no difference in resting membrane potentials with or without nicotine treatment between sham and T2DM CVP neurons (Figure 2F). Taken together, these data indicate that CVP neurons exhibited attenuated neuronal functionality in T2DM rats.

### Uncoupling protein 2 (UCP2) expression was reduced in T2DM

**FIGURE 3:**
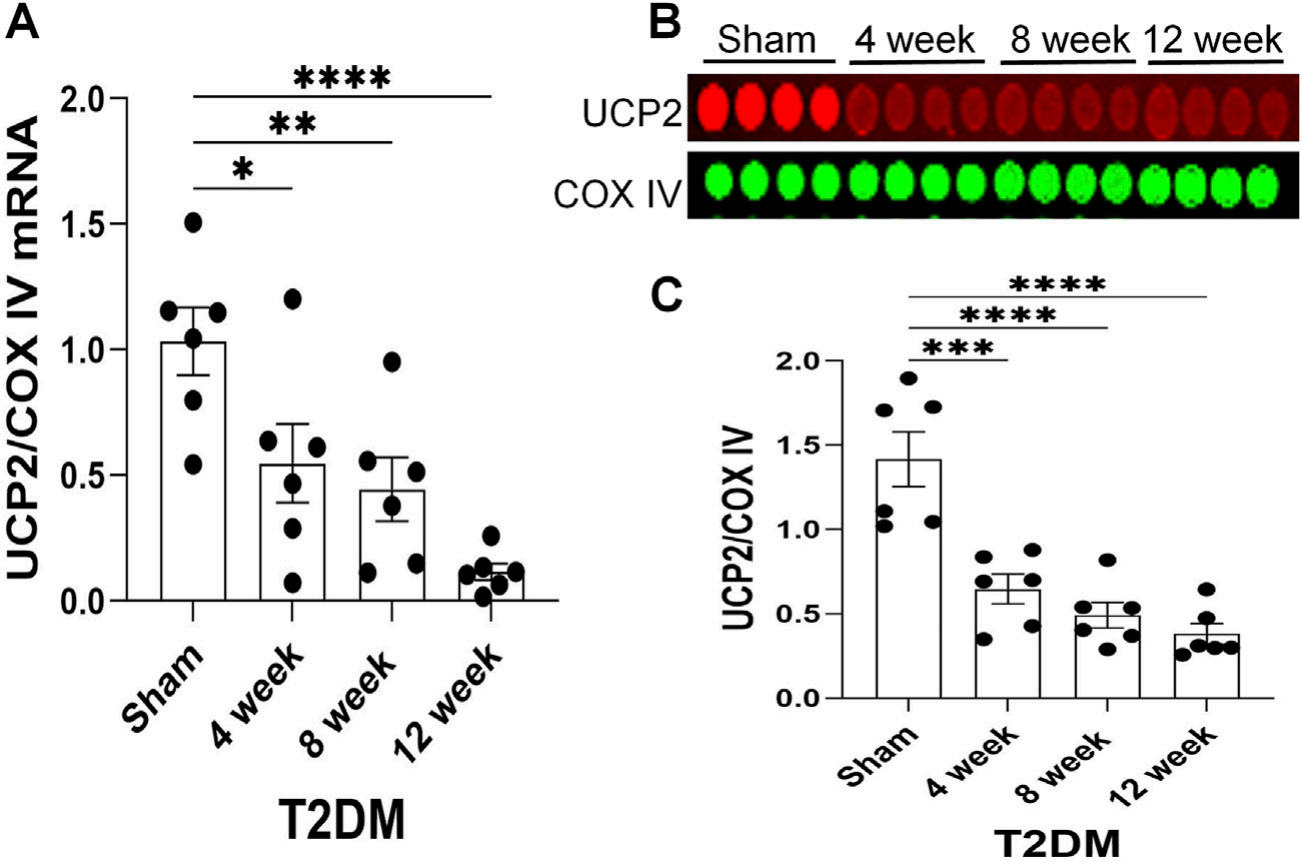
A time course of UCP2 expression as T2DM develops. **(A)** Mean RT-qPCR data of UCP2 transcript expression relative to cytochrome c oxidase subunit IV (COX IV, mitochondrial maker) transcript expression in sham, 4-week, 8-week, and 12-week post-T2DM ICG; n = 6/group. **(B, C)** Representative image **(B)** and mean data **(C)** of RPPA analysis of UCP2 and COX IV protein expression in sham, 4-week, 8-week, and 12-week post-T2DM ICG; n = 6/group. *P < 0.05, **P < 0.01, ***P < 0.001, ****P < 0.0001 vs. sham.

**FIGURE 4:**
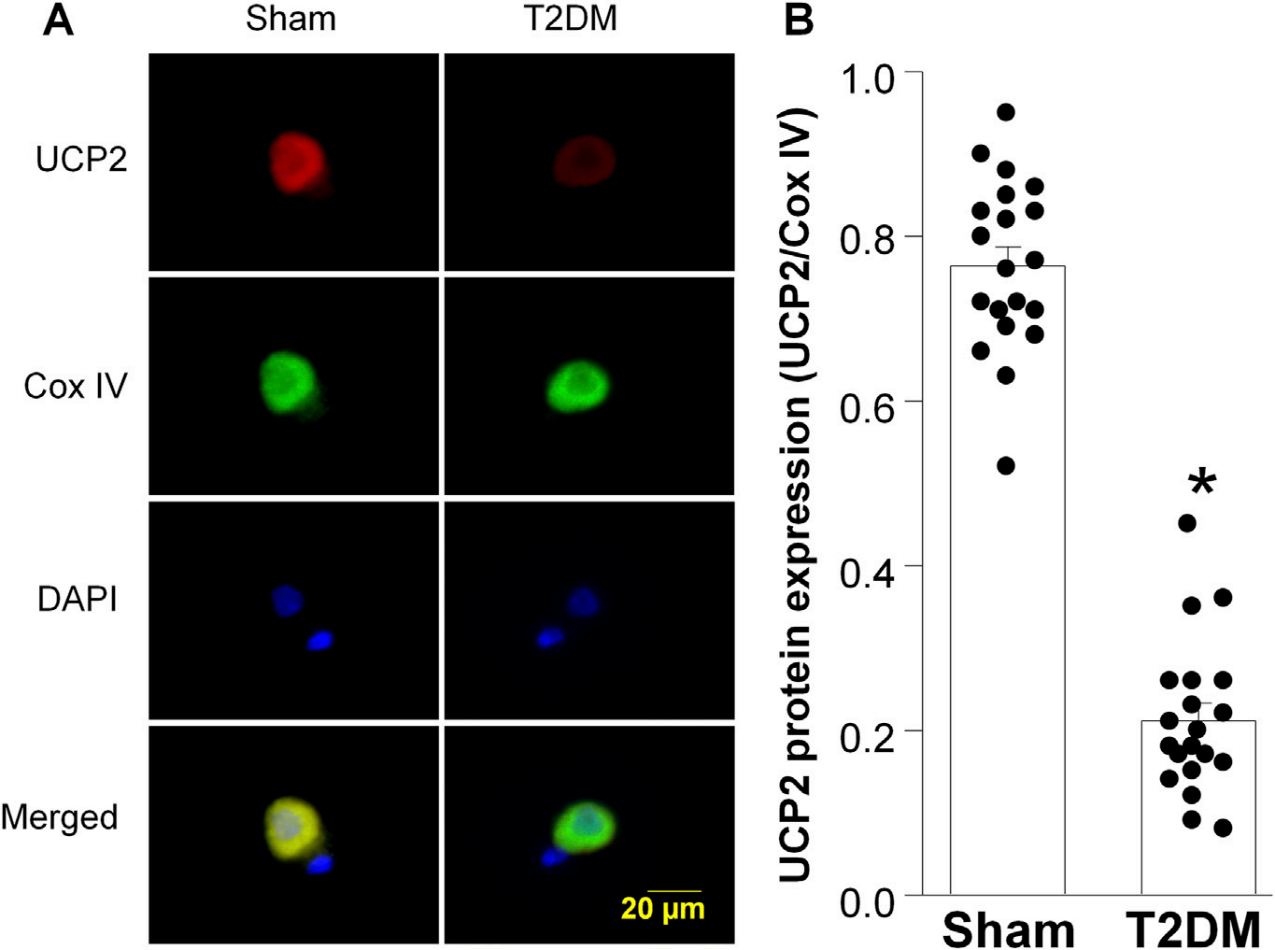
Uncoupling protein 2 (UCP2) expression in CVP neurons from sham and type 2 diabetic (T2DM) rats. **(A)** Representative images showing UCP2 (red color), cytochrome c oxidase subunit IV (Cox IV, mitochondrial marker, green color), 4,6-diamidino-2-phenylidole (DAPI, cell nucleus marker, blue color), and colocalization of UCP2 and Cox IV in mitochondria. **(B)** Quantitative data for protein expression of UCP2 in mitochondria. Data are mean ± SEM; n = 20 neurons in each group. *P < 0.05 vs. sham.

Along with diminished nAChR current density, our previous studies have reported that T2DM reduces voltage-gated calcium current density in CVP neurons through reducing the expression of N-type calcium channels (Liu et al., 2012). Additionally, the alteration in N-type calcium channel expression was revealed to result from elevated levels of hydrogen peroxide (H_2_O_2_, a type of reactive oxygen species (ROS)) (Liu et al., 2012; Zhang et al., 2022). As the mitochondria is a primary source of intracellular ROS (Hernansanz-Agustín and Enríquez, 2021), we examined the impact of T2DM on the expression of Uncoupling protein 2 (UCP2), a member of the mitochondrial uncoupling protein family, which exerts antioxidant properties and downregulates oxidative stress (Jabůrek et al., 2013; Forte et al., 2021). Upon examination of UCP2 expression as T2DM progresses, RT-qPCR and RPPA analysis revealed that the expression of UCP2 mRNA and protein in T2DM ICGs was attenuated as early as 4 weeks post-T2DM induction and gradually decreased through 12-week T2DM (Figure 3). Additionally, the data from immunofluorescence staining also revealed that 12-week T2DM significantly lowered the levels of UCP2 in CVP neurons as compared to sham (Figures 4A,B).

### Development of leptin resistance in T2DM ICG

**FIGURE 5:**
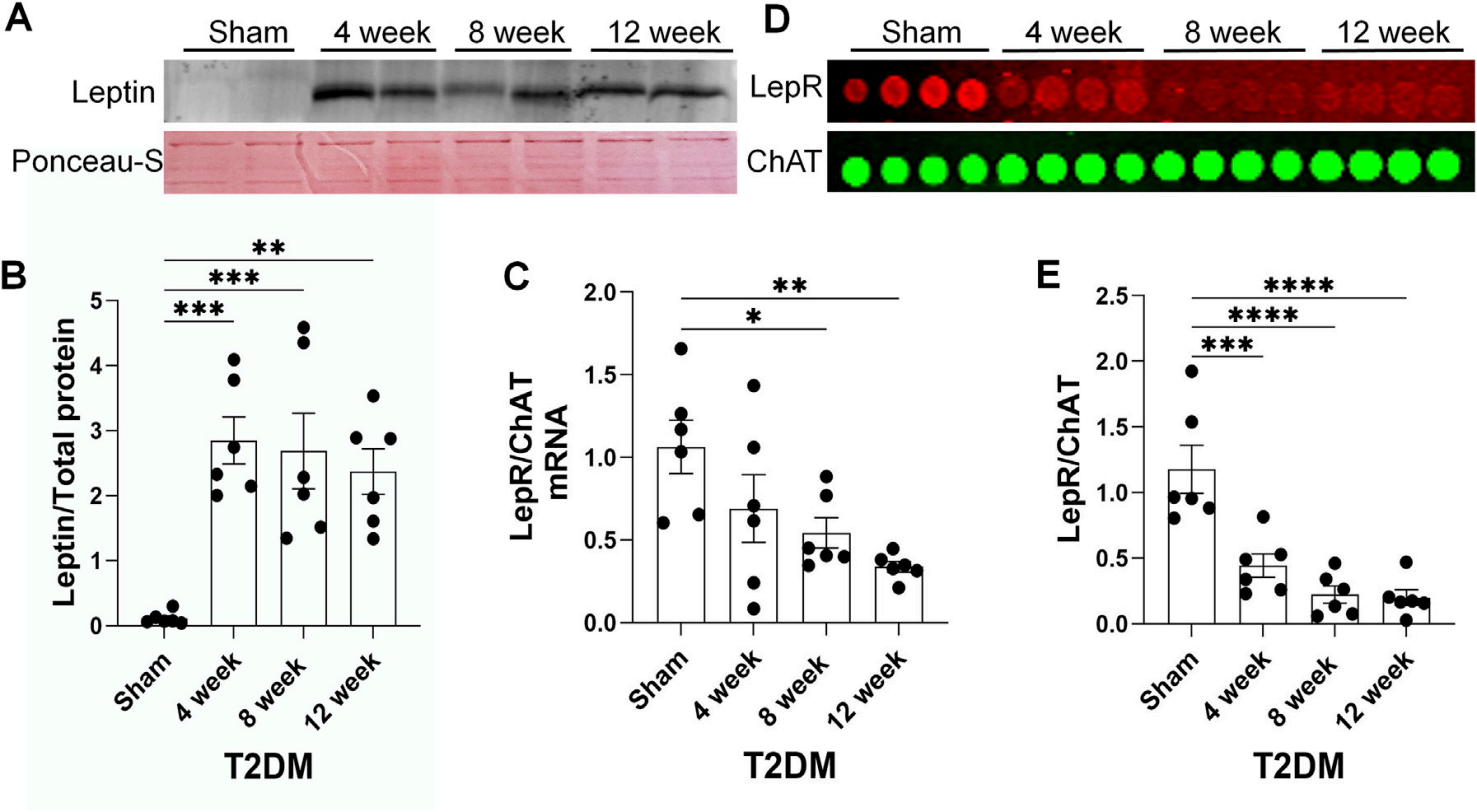
A time course of leptin resistance as T2DM develops. **(A, B)** Representative image **(A)** and mean data **(B)** of Western blot analysis of Leptin expression and ponceau-S total protein stain (housekeeping) in epicardial adipose pads from sham, 4-week, 8-week, and 12-week post-T2DM; n = 6/group. **(C)** Mean RT-qPCR data of lepR transcript expression relative to choline acetyltransferase (ChAT, cholinergic neuronal marker) transcript expression in sham, 4-week, 8-week, and 12-week post-T2DM ICG; n = 6/group. **(D, E)** Representative image **(D)** and mean data **(E)** of RPPA analysis of lepR and ChAT protein expression in sham, 4-week, 8-week, and 12-week post-T2DM ICG; n = 6/group. *P < 0.05, **P < 0.01, ***P < 0.001, ****P < 0.0001 vs. sham.

Leptin resistance is a common pathology associated with T2DM development, characterized by attenuated responses to the hormone leptin, despite elevated leptin levels (Gruzdeva et al., 2019; Knight et al., 2010). As leptin has previously been shown to drive UCP2 expression (Ho et al., 2010), we examined the impact of leptin resistance in CVP neurons. In this study, leptin resistance was defined as exhibiting reduced levels of leptin receptor (lepR) expression in CVP neurons despite elevated levels of leptin protein. As our previous study reported 12-week T2DM rats exhibit hyperleptinemia (Liu et al., 2012), we examined leptin expression in epicardial adipose pads as T2DM developed. Western blot analysis revealed that levels of leptin significantly increased in the epicardial adipose pads as early as 4 weeks post-T2DM induction and remained elevated through 12-week T2DM, as compared to sham (Figures 5A,B). To determine if elevated leptin levels could be a result of a T2DM induced increase in white epicardial adipose, we visually inspected isolated rat hearts and found that 12-week T2DM rat hearts appeared to have more white epicardial adipose tissue as compared to sham (Supplementary Figure 2).

**FIGURE 6:**
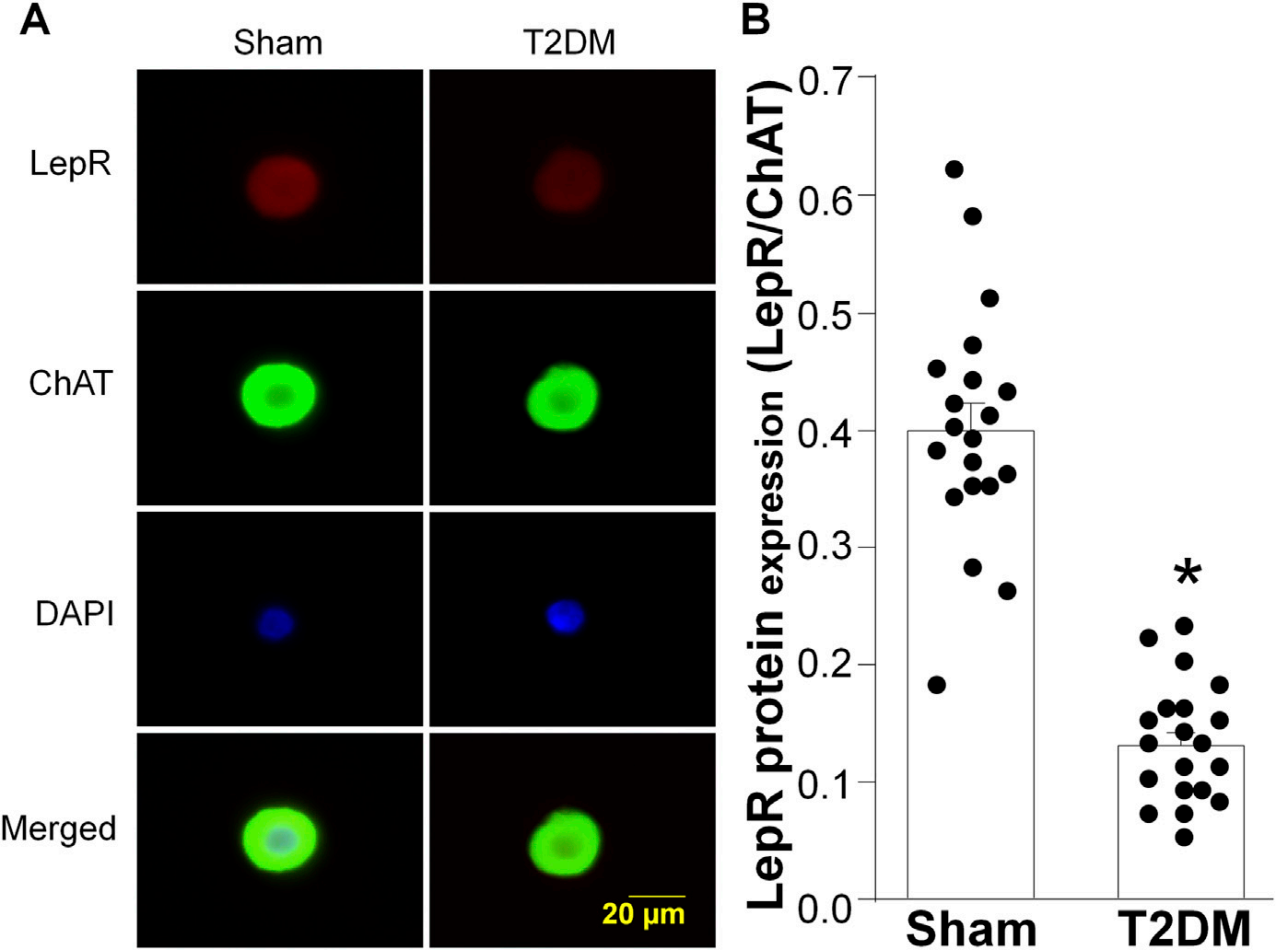
Protein expression of leptin receptors (lepR) in CVP neurons from sham and type 2 diabetic (T2DM) rats. **(A)** representative images showing lepR (red color), choline acetyltransferase (ChAT, a cholinergic neuronal marker, green color), 4,6-diamidino-2-phenylidole (DAPI, cell nucleus marker, blue color), and colocalization of lepR and ChAT in CVP neurons. **(B)** quantitative data for protein expression of lepR in CVP neurons. Data are mean ± SEM; n = 20 neurons in each group. *P < 0.05 vs. sham.

As T2DM epicardial adipose pads exhibited elevated levels of leptin, we examined lepR expression in rat ICG to determine if T2DM ICG experience leptin resistance. RT-qPCR and RPPA analyses showed that ICG lepR mRNA and protein levels were reduced 4 weeks post-T2DM induction and were continually kept at a low level through 12-week T2DM (Figures 5C–E). Additionally, immunofluorescent data also revealed a reduced level of lepR protein in the CVP neurons from 12 weeks post-T2DM rats compared to sham rats (Figures 6A,B). Taken together, these data reveal that leptin resistance develops in T2DM ICGs, which may contribute to CVP neuronal dysfunction in T2DM.

## Discussion

In the present study, we found that HFD-STZ-induced T2DM reduced cardiac parasympathetic activity, which might be partially attributed to functional remodeling of CVP neurons located in the ICG. Additionally, leptin resistance possibly correlates with the functional remodeling of CVP neurons through the alteration of UCP2 in T2DM. These findings suggest that leptin resistance could be a therapeutic target to improve CVP neuronal function and cardiac vagal activity in T2DM.

### T2DM rat model

T2DM is a complex disease whose development and pathological progression is the result of synergism of many factors including nutrition, environment, and genetics (Wu et al., 2014). In the early stages of T2DM, patients develop insulin resistance. To combat this, pancreatic β-islet cells secrete greater amounts of insulin to keep homeostatic blood glucose levels (Rachdaoui, 2020; Wondmkun, 2020). As T2DM progresses, this compensatory release of insulin diminishes in effectiveness, leading to reduced glucose tolerance and a hyperglycemic state (Zheng et al., 2018). In addition to insulin resistance and hyperglycemia, individuals with T2DM often develop other pathophysiologies, such as dyslipidemia, hyperleptinemia, and increased visceral adipose accumulation (Mooradian, 2009; Hulkoti et al., 2022; Zhao and Li, 2022). The heterogeneity in the pathogenesis and complications experienced by diabetic patients makes identifying an experimental animal model that mimics the progression and clinical characteristics of human T2DM a significant challenge in studying T2DM.

Using the same rat T2DM model, in our previous study we found that T2DM rats exhibited reduced insulin sensitivity, elevated triglyceride levels, hyperleptinemia, increased visceral white adipose, and diminished brown adipose (Liu et al., 2012). Similar changes in insulin sensitivity, triglyceride levels, and blood glucose levels have also been reported in other studies using this model (Hussein et al., 2012; Khan et al., 2013; Cui et al., 2018). While the T2DM model used in this study has been reported to mimic clinical T2DM pathologies (hyperglycemia, dyslipidemia, etc.) (Srinivasan et al., 2005; Skovsø, 2014; Liu et al., 2012), we acknowledge that the possible effects that HFD-induced obesity or STZ treatment alone have on CVP neuronal function and cardiac parasympathetic activity cannot be excluded.

### Baroreflex sensitivity and functional remodeling of CVP neurons in T2DM

As cardiovascular events are the leading cause of death in T2DM patients (Walker and Cubbon, 2015; Wong and Sattar, 2023), understanding the progression of cardiovascular dysfunction in T2DM is of the utmost importance in the effort to decrease T2DM mortality. A wide array of evidence in clinical studies and animal experiments has shown that diminished arterial baroreflex sensitivity is a common complication in T2DM (Kim et al., 2019; Gupta et al., 2020; Samora et al., 2023; Hamaoka et al., 2024). The baroreflex function is regulated at multiple integrative centers including the baroreceptors at the afferent limb, central components, and autonomic efferent components (Suarez-Roca et al., 2021). Dysregulation of any of these branches could contribute to T2DM-induced cardiac autonomic dysfunction. For example, studies report that baroreceptor neurons in the nodose ganglia in type 1 diabetic rats exhibit altered neuronal excitability (Tu et al., 2010; da Silva-Alves et al., 2023). It is certainly possible that changes in sensory afferent signaling in nodose ganglia could contribute to the diminished baroreflex sensitivity we observed in this study.

Autonomic efferent control of the baroreflex includes both sympathetic components as well as parasympathetic components (Durães Campos et al., 2018; Suarez-Roca et al., 2021). Diminished baroreflex sensitivity could result from an increase in cardiac sympathetic signaling to the heart, a reduced parasympathetic signaling to the heart, or a combination of both effects (Huggett et al., 2003; Thaung et al., 2015; Young et al., 2019). This study focused on the impact of parasympathetic decline in baroreflex sensitivity because the decline in parasympathetic tone predates sympathetic overactivation in T2DM (Hadad et al., 2022; Rasmussen et al., 2022). Parasympathetic cardiac autonomic tone is regulated through the activity of preganglionic neurons in the DVMN and postganglionic CVP neurons in the ICG (Durães Campos et al., 2018). One previous study reported that isolated parasympathetic DVMN neurons depolarize in response to leptin (Li et al., 2007). As DVMN neurons activate CVP neurons, it is possible that diminished preganglionic cardiac parasympathetic neuronal activity, in response to DVMN neurons becoming resistant to leptin, could contribute to T2DM induced cardiac parasympathetic withdrawal. Similarly, our previous studies have reported that T2DM CVP neurons exhibit reduced functionality resulting in compromised cardiac parasympathetic function (Liu et al., 2012; Liu et al., 2015; Zhang et al., 2022).

In this study, we confirm that nAChR currents are reduced in T2DM CVP neurons and further found that the reduced nAChR currents could contribute to the decrease in CVP neuronal excitability, evidenced by the reduced frequency of action potentials. These data support that T2DM-induced alterations in baroreflex sensitivity is partially due to reduced cardiac parasympathetic activity at the level of the ICG. Nevertheless, the potential role of cardiac sympathetic overactivation in T2DM-induced cardiovascular autonomic dysfunction cannot be discounted. Previous studies have reported that T2DM patients exhibit sympathetic hyperactivity (Huggett et al., 2003; Young et al., 2019), which could contribute to ventricular arrhythmogenicity (Albarado-Ibañez et al., 2013; Gardner et al., 2016). Furthermore, one study identified increased sympathetic nerve activity in the stellate ganglion, which regulates sympathetic cardiac autonomic activity, of Zucker fatty acid rats (Thaung et al., 2015). As such, the symapathovagal balance must be considered when assessing changes in cardiac autonomic function in T2DM.

### Potential molecular mechanisms leading to functional remodeling of CVP neurons in T2DM

As altered CVP neuronal excitability has been reported to contribute to cardiac vagal dysfunction and malignant ventricular arrhythmogenesis in T2DM (Zhang et al., 2022), understanding the molecular processes that drive CVP neuronal dysfunction is paramount in the effort to eradicate cardiac autonomic dysfunction in T2DM patients. Previous studies showed that reactive oxygen species (ROS) induce deleterious functional changes in multiple neuronal cell types in T2DM, including CVP neurons (Zherebitskaya et al., 2009; Liu et al., 2014; Zhang et al., 2022; Zhang et al., 2023). One previous study reported that T2DM elevated the levels of H_2_O_2_ (a type of ROS) and reduced the expression of the ROS scavenger catalase in CVP neurons, which contributed to CVP neuronal dysfunction (Zhang et al., 2022). Similarly, induction of oxidative stress in ICG neurons via H_2_O_2_ stimulation alters calcium and potassium currents and reduces neuronal excitability (Whyte et al., 2009). Additionally, decreasing ROS levels in CVP neurons through reestablishing antioxidant scavengers, such as catalase and superoxide dismutase, has been shown to improve CVP neuronal function (Whyte et al., 2009; Zhang et al., 2022). Although the driver of ROS accumulation in T2DM CVP neurons has not yet been elucidated, T2DM-impaired mitochondria could be a significant source of intracellular ROS (Kizhakekuttu et al., 2012; Fiorentino et al., 2021; Hernansanz-Agustín and Enríquez, 2021).

Our present study explored the impact of T2DM on UCP2, a mitochondrial ROS regulator. Normally, UCP2 functions in the maintenance of mitochondrial membrane potential and regulates mitochondrial oxidative stress through its activity to dissipate the proton gradient at the inner mitochondrial membrane (Nesci and Rubattu, 2024). Our current study demonstrated that the expression of UCP2 protein and mRNA was reduced in CVP neurons early in the development of T2DM in HFD-STZ T2DM rats (4 weeks post-T2DM induction), which is consistent with a previous study that reported reduced UCP2 expression in kidney tissue of T2DM patients (de Souza et al., 2015). Diminished UCP2 expression has been associated with increased cellular oxidative stress in a rat brain microvascular endothelial cell line, a rat renal proximal tubular endothelial cell line, and the brain tissue from a spontaneously hypertensive rat model (Rubattu et al., 2017; Forte et al., 2021). Upregulation of UCP2 expression by treatment of perindopril has been shown to rescue attenuated mitochondrial membrane potential in a rat model of diabetic retinopathy (Zheng et al., 2009). Furthermore, re-establishment of UCP2 expression in a UCP2 knockout mouse model decreased ROS accumulation and NLRP3 inflammasome activation in mouse astrocytes (Du et al., 2016). These studies, including our present and previous data (Zhang et al., 2022; Zhang et al., 2023) make a strong argument that restoring UCP2 expression may serve as a potential therapeutic strategy for improving T2DM-induced CVP neuronal dysfunction.

To further explore potential mechanisms that lead to reduction of UCP2 expression, we examined the impact of leptin resistance on T2DM CVP neurons. Canonically, leptin binds leptin receptors expressed on the cell surface and initiates the Janus kinase/signal transducer and activator of transcription (JAK-STAT) pathway to exert cellular transcriptional regulation (Procaccini et al., 2009), though activation of other signaling molecules, such as PKC and PKA, has also been reported (Barrenetxe et al., 2004). Previous studies have reported that leptin/lepR signaling induced an increase in UCP2 expression in SH-SY5Y neuronal cells and white adipose tissue of Sprague-Dawley rats (Scarpace et al., 1998; Ho et al., 2010). Furthermore, Lapp et al. reported that STAT3 could directly bind the UCP2 promoter to drive UCP2 expression (Lapp et al., 2014). These studies make a strong argument that changes in leptin/LepR signaling, such as the development of leptin resistance, during T2DM development may be a regulator of UCP2 expression.

Leptin resistance often develops as a result of T2DM (Bidulescu et al., 2020; Manglani et al., 2024; Vilariño-García et al., 2024), and we have previously reported that T2DM rats exhibit hyperleptinemia (Liu et al., 2012). However, leptin resistance may not impact all tissues, but develops selectively, which is a novel concept of selective leptin resistance (Correia et al., 2002; Mark et al., 2002). One previous study has shown that, in agouti yellow obese mice, leptin’s impact on renal sympathoexcitation was preserved, despite the loss of leptin’s weight reducing actions (Correia et al., 2002). Similarly, Munzberg et al. reported that neurons in the arcuate nucleus, but not the ventromedial hypothalamus, or dorsal medial hypothalamus exhibit leptin resistance in diet-induced obese mice (Münzberg et al., 2004).

To ascertain the state of leptin resistance in T2DM ICG, we first examined the state of leptin expression in epicardial adipose pads and found that T2DM epicardial adipose pads express elevated levels of leptin as early as 4 weeks post-T2DM induction. Upon visual examination of our rat hearts, we identified that T2DM rats appeared to have more white epicardial adipose as compared to sham (Supplementary Figure 2). This increase in epicardial leptin may be due to an increase in white epicardial adipose tissue, as previous studies have shown that white adipose tissue secretes more leptin than brown adipose (Moinat et al., 1995; Cinti et al., 1997). Examination of lepR expression in CVP neurons revealed T2DM induces reduced levels of lepR as early as 4 weeks post-T2DM induction, and levels stay diminished as T2DM progresses. The combination of elevated leptin levels and reduced leptin receptor expression indicates that CVP neurons in the ICG develop leptin resistance during the early stages of T2DM development. So far, we do not know whether leptin resistance in CVP neurons is a form of selective leptin resistance or if the rats experience a global leptin resistance in this T2DM rat model, which should be clarified in future studies. To our knowledge, we are the first to report that CVP neurons develop leptin resistance through reductions of lepR expression in T2DM.

As T2DM CVP neurons exhibit leptin resistance, we expect that lepR controlled intracellular signaling will become dysregulated in T2DM. As leptin/lepR signaling has previously been implicated in intracellular ROS maintenance (Bilbao et al., 2015; Frühbeck et al., 2017), and one previous study in adipose tissues of T2DM mice reported that restoration of canonical leptin/lepR signaling attenuated intracellular ROS accumulation (Frühbeck et al., 2017), it is certainly possible the leptin resistance-UCP2-ROS signaling pathway might be associated with CVP neuronal dysfunction in T2DM. It is important to acknowledge that leptin resistance is not the only possible explanation for CVP neuronal dysfunction. We have previously reported that 12-weeek T2DM rats exhibit reduced insulin sensitivity/insulin resistance (Liu et al., 2012). It may be that CVP neuron dysfunction is also driven by reduced insulin responsiveness. One previous study correlated the development of insulin resistance in human patients with diminished cardiac vagal tone (Ziegler et al., 2018). Similarly, insulin resistance in sensory dorsal root ganglion neurons has been shown to alter neuronal function through dysregulated Akt activation (Grote et al., 2013). As leptin resistance and insulin resistance occur simultaneously in T2DM, it is also certainly possible that a synergism of diminished leptin and insulin response leads to reduced CVP neuronal function. We, of course, acknowledge that our present study has not shown the direct association among leptin resistance, UCP2, and CVP neuronal function in T2DM, which need further studies to be definitively elucidated, such as *in vivo* overexpression of lepR or UCP2 in CVP neurons.

In conclusion, the data presented here identified cardiovascular parasympathetic dysfunction in HFD-STZ T2DM rats, which is partially attributed to functional remodeling of CVP neurons during T2DM development. Additionally, leptin resistance and reduced UCP2 expression initiated in the early stage of T2DM might be potential triggers to cause CVP neuronal dysfunction, and further result in attenuation of cardiovascular parasympathetic activation and cardiac arrhythmogenesis during the progression of T2DM. These triggers should be potential therapeutic targets to improve the prognosis of T2DM patients.

## Data Availability

The original contributions presented in the study are included in the article/Supplementary Material, further inquiries can be directed to the corresponding author.
