# Supplementary material for: Altered leptin signaling and attenuated cardiac vagal activity in rats with type 2 diabetes
Source: Front Physiol. 2025 Feb 26;16:1547901. doi: 10.3389/fphys.2025.1547901 (PMC11897569; doi:10.3389/fphys.2025.1547901)
Supplement: Supplementary file 1 [file Table1.docx]

Supplementary Material

**Supplemental Table 1**. Metabolic characteristics of sham and T2DM rats

|  | Body weight (g) | Blood glucose (mg/dl) |
| --- | --- | --- |
| Sham (n=24) | 406.4 ± 9.9 | 98.3 ± 2.4 |
| 4w T2DM (n=16) | 364.6 ± 8.4 | 93.3 ± 3.3 |
| 8w T2DM (n=16) | 386.3 ± 8.5 | 387.3 ± 19.7* |
| 12w T2DM (n=24) | 414.0 ± 8.9 | 441.8 ± 11.5* |

Data are means ± SEM. Statistical significance was determined by one-way ANOVA. *P<0.05 vs. sham. 4w, 8w, and 12w: 4, 8, and 12 weeks. T2DM: Type 2 diabetes mellitus.

| **Target** | **Abbreviation** | **Application** | **Dilution** | **Company** | **Catalog number** |
| --- | --- | --- | --- | --- | --- |
| Leptin Receptor | LepR | IF | 1:100 | Santa Cruz | Sc-8391 |
| Choline acetyltransferase | ChAT | IF | 1:100 | Millipore | AB144P |
| Cytochrome c oxidase subunit IV | COX IV | IF | 1:200 | Abcam | Ab16056 |
| Uncoupling protein 2 | UCP2 | IF | 1:50 | LS Bio | LS-B3249 |
| Leptin Receptor | LepR | RPPA | 1:1000 | Protein Tech | 20966-1-AP |
| Choline acetyltransferase | ChAT | RPPA | 1:1000 | Protein Tech | 20747-1-AP |
| Cytochrome c oxidase subunit IV | COX IV | RPPA | 1:1000 | Abcam | ab16056 |
| Uncoupling protein 2 | UCP2 | RPPA | 1:1000 | Protein Tech | 11081-1-AP |
| Leptin |  | Western | 1:5000 | Peprotech | 500-P185G |

**Supplemental table 2**. Antibodies used in immunofluorescence microscopy, western blot, and RPPA. IF: Immunofluorescence microscopy, RPPA: Reverse Phase Protein Microarray, Western: Western blot

| **Accession Number** | **Sequence (5'->3')** | **Target** |
| --- | --- | --- |
| NM_012596.3 | GTCAACTACGCTCTTCTGATG | LepR |
| NM_012596.3 | TCATCTGTGACTTCCATACG | LepR |
| NM_001170593.1 | TCTGCTGTTATGGACCCGTG | ChAT |
| NM_001170593.1 | GCAAACTCCACAGACGAGGT | ChAT |
| NM_019354.3 | TTGTCAACTGTACTGAGCTG | UCP2 |
| NM_019354.3 | CAGAAGTGAAGTGGCAAG | UCP2 |
| NM_017202.1 | TCTACTTCGGTGTGCCTTCG | COX IV |
| NM_017202.1 | CCACATCAGGCAAGGGGTAG | COX IV |
|  |  |  |

**Supplemental table 3**. Primers used in RT-qPCR analysis.


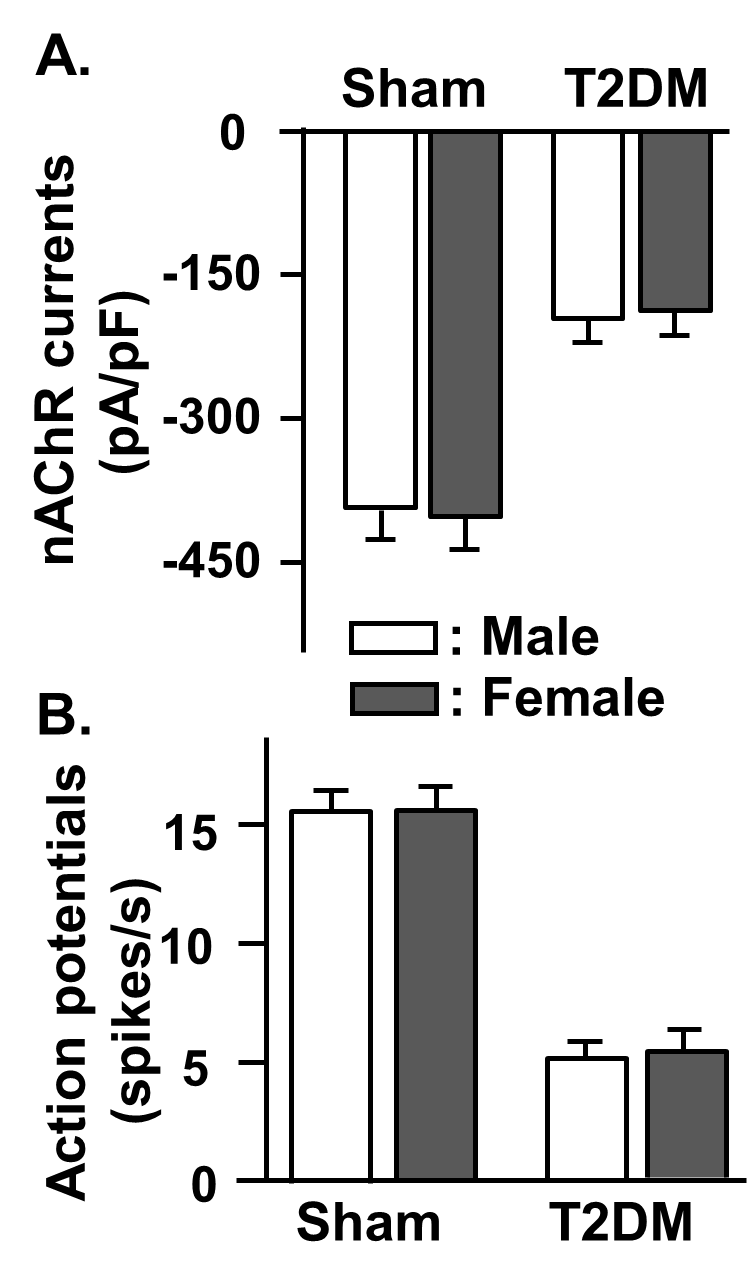


**Supplementary Figure 1**. nAChR currents and cell excitability in CVP neurons from male and female rats. nAChR currents **(A)** and action potentials **(B)** in cardiac vagal neurons from male and female sham and T2DM rats. Data are mean ± SEM; N=8 neurons/group.


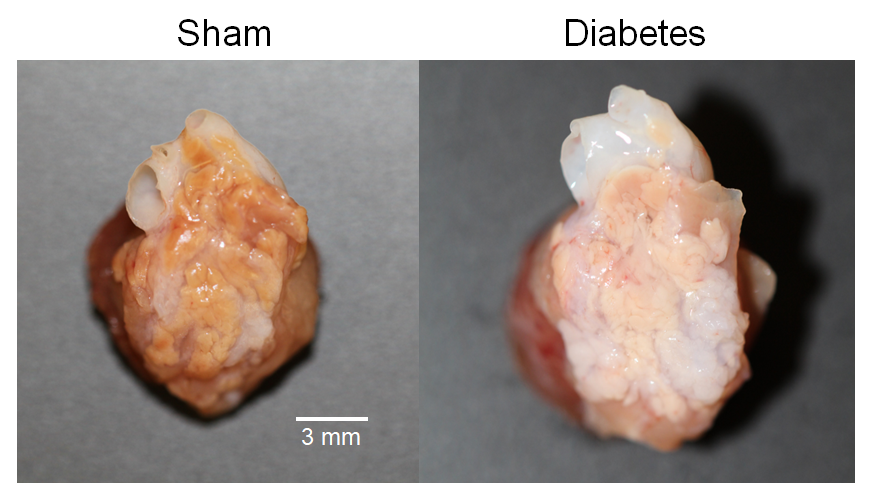


**Supplementary Figure 2.** Epicardial adipose of T2DM and sham rat hearts. A representative image showing epicardial adipose of sham (left) and T2DM (right) rat hearts.
